# Isolation and Genetic Characterization of Rift Valley fever virus from Aedes vexans arabiensis, Kingdom of Saudi Arabia

**DOI:** 10.3201/eid0812.020194

**Published:** 2002-12

**Authors:** Barry R. Miller, Marvin S. Godsey, Mary B. Crabtree, Harry M. Savage, Yagob Al-Mazrao, Mohammed H. Al-Jeffri, Abdel-Mohsin M. Abdoon, Suleiman M. Al-Seghayer, Ali M. Al-Shahrani, Thomas G. Ksiazek

**Affiliations:** * Centers for Disease Control and Prevention, Atlanta, Georgia, USA; † Ministry of Health, Riyadh, Kingdom of Saudi Arabia

**Keywords:** Rift Valley fever, Rift Valley fever virus, Saudi Arabia, Aedes vexans arabiensis

## Abstract

An outbreak of Rift Valley fever in the Kingdom of Saudi Arabia and Yemen in 2000 was the first recognized occurrence of the illness outside of Africa and Madagascar. An assessment of potential mosquito vectors in the region yielded an isolate from Aedes vexans arabiensis, most closely related to strains from Madagascar (1991) and Kenya (1997).

On September 10, 2000, accounts of unexplained hemorrhagic fever in humans and associated illness in livestock along the southwestern border of Saudi Arabia and neighboring Yemen were reported to the Ministry of Health, Kingdom of Saudi Arabia. On September 15, 2000, the Centers for Disease Control and Prevention (CDC), Atlanta, Georgia, confirmed a diagnosis of Rift Valley fever in serum samples submitted by the Ministry of Health. This confirmation marked the first occurrence of Rift Valley fever outside of Africa (1).

Rift Valley fever virus (RVFV) is an important veterinary pathogen in Africa causing abortions and deaths in young animals, primarily goats and sheep (2). This mosquito-borne virus can also infect humans by arthropod blood-feeding or by contact with infected animal fluids and tissues. RVFV infection in humans is generally not apparent or is self-limiting; serious complications including hemorrhagic fever, encephalitis, and retinitis can occur. Vector-borne virus transmission in Africa is generally associated with periodic heavy rainfall during epizootics and attendant human infections (2).

We conducted an entomologic investigation in the Asir, Jizan, and Makkah Regions, north of the suspected origin of the outbreak in Jizan, and in neighboring Yemen. Because this outbreak in the Arabian Peninsula was the first recorded outside of Africa, we wanted to determine the potential arthropod vectors and their larval habitats. A review of recent human infections indicated that the affected households were located in the foothills and at the base of the Sarawat Mountains. Four locations were selected for arthropod collections, Muhayil, Al Birk, Rijal Alma'a, and Al Majardah, because these areas are representative of the different ecologic habitats from coastal plain to mountainous regions. This fertile plain used for cultivation is known as the Tihamah.

## The Study

We collected adult and immature arthropods on December 5–13, 2000, using carbon dioxide–baited CDC miniature light traps and by sampling potential larval habitats with dippers. Adult specimens were frozen in liquid nitrogen for later virus testing in Fort Collins, Colorado. To investigate potential vertical transmission of RVFV, mosquito larvae were reared to adults in the malaria control laboratory in Abha, Asir Region, for later virus testing in the United States.

All collection sites were in very arid habitats; the soil is dry and rocky, and Acacia species are the only trees present. Livestock, including goats (the predominant animal), sheep, camels, and cattle, were present at every site. In general, livestock are housed at night very close to the owners’ homes. Light traps were hung in and near the residences of recent patients, and the area was examined for larval habitats. Typical larval habitats included wastewater catchments from houses that yielded mainly Culex pipiens complex mosquitoes; pools at the edge of wadis; and small, walled, passively or actively flooded cultivated plots that yielded Aedes vexans arabiensis and Ae. vittatus (3). Ae. (Stegomyia) unilineatus, a mosquito species previously recorded from Africa, India, and Pakistan, was found in light trap collections from several sites (Godsey MS, submitted for publication). The immature and adult arthropod collections are presented in Table 1. The low species diversity and small numbers collected in light traps may have reflected that rainfall was light to nonexistent 2 weeks before our collection efforts. We did examine one walled farming plot, which contained water to a depth of approximately 5 cm; it held enormous numbers of Ae. vexans arabiensis larvae and pupae. Overnight, this habitat had dried up to a 2-m diameter pool.

**Table 1:** Arthropods collected in the Kingdom of Saudi Arabia, December 5–13, 2000

Taxon	Female	Male
Specimens reared from larvae and pupae		
*Aedes vexans arabiensis*	887	858
*Ae*. *vittatus*	0	2
*Culex pipiens* complex	67	47
Specimens collected in carbon dioxide–baited light trap		
*Anopheles dthali*	50	0
*Ae. vexans arabiensis*	122	0
*Ae. vittatus*	6	3
*Ae. aegypti*	2	0
*Ae. unilineatus*	18	3
*Cx. pipiens* complex	266	149
*Cx. nebulosus*	1	0
*Cx. salisburiensis*	1	0
*Cx. tritaeniorhynchus*	42	0
*Aedes species*	16	0
*Anopheles* species	142	1
*Culex* specie*s*	31	0
*Psychodidae*	61	18
*Ceratopogonidae*	26	3

Collected arthropod specimens were identified and placed into pools of up to 50 individual mosquitoes by collection site. A total of 161 pools were triturated, clarified by centrifugation, and spread onto confluent sheets of Vero cells in six-well plates (4). A single pool (SA01 #1322) yielded a virus isolate. The virus was identified as RVFV by sequencing a DNA fragment amplified from the M segment by reverse transcription-polymerase chain reaction using the primers RVF3082 5′actttgtgggagcagccgtatctt3′ and RVF3400 5′cctgcttcccgcctatcatcaaat3′.

RVFV was isolated from a pool of 37 Ae. vexans arabiensis female mosquitoes collected by light trap at site 3 (N18° 45.089 min; E41° 56.373 min) near the city of Muhayil. A human infection was recorded from this site ("Agida"); we also witnessed aborted bovine fetuses on the property. Other arthropods collected at this site included Cx. tritaeniorhynchus and psychodid sandflies.

RVFV is a member of the virus family, Bunyaviridae; the genomes of these viruses exist in three pieces or segments: small (S), medium (M), and large (L). We sequenced a portion of each of the genome segments and analyzed them by the maximum likelihood algorithm in PAUP (5) in relation to the published sequences of other geographic isolates to determine the possible origin of the mosquito isolate and whether the isolate was a possible reassortant between two existing virus strains (5,6). Maximum likelihood trees for each genomic segment shared identical topologies (data not shown). The congruence of placement of the Saudi virus strain in the three trees indicated that this virus was not a reassortant. The phylogram for segment M (Figure; Table 2) demonstrates that the most closely related RVFV isolates were from Kenya (1997) and Madagascar (1991). Reasonable hypotheses to explain how RVFV was introduced into the Kingdom of Saudi Arabia (and/or Yemen) from East Africa are that an infected mosquito was carried over the narrow waterway between the Red Sea and the Gulf of Aden by air currents or that infected livestock were imported from East Africa. How long the virus was in the Arabian Peninsula before the epidemic occurred is unknown.

**Figure:**
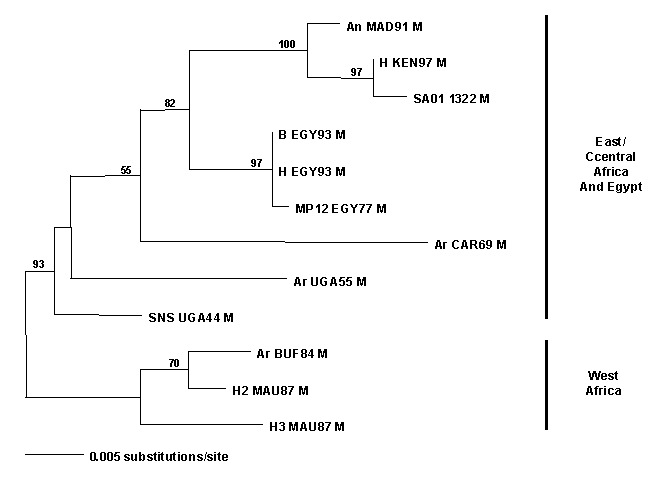
Maximum likelihood phylogram of African *Rift Valley fever virus* strains (see Table 2) and mosquito isolate from the Kingdom of Saudi Arabia based on a 655-bp DNA fragment from the M segment (4).

**Table 2:** *Rift Valley fever virus* strains used in the phylogenetic analyses

Virus designation	Strain name	Geographic origin	Year of isolation	Source
SNS UGA44	Smithburn	Uganda	1944	Entebbe strain
Ar UGA55	Lunyo	Uganda	1955	Mosquito
Ar CAR69	Ar B 1976	Central African Rep.	1969	Mosquito
MP12 EGY77	MP12	Egypt	1977	ZH 548 strain
Ar BUF84	Ar D 38457	Burkina Faso	1984	Mosquito
H2 MAU87	H D 47311	Mauritania	1987	Human
H3 MAU87	H D 47408	Mauritania	1987	Human
An MAD91	An Mg 990	Madagascar	1991	Bovine
B EGY93	B EGY 93	Egypt	1993	Buffalo
H EGY93	H EGY 93	Egypt	1993	Human
H KEN97	384-97.1	Kenya	1997	Human
Ar SA01	SA01 1322	Saudi Arabia	2001	Mosquito

The most abundant culicine mosquitoes we collected were Ae. vexans arabiensis, Cx. pipiens complex, and Cx. tritaeniorhynchus. All three species should be considered important epidemic and epizootic vectors of RVFV in Saudi Arabia. The floodwater mosquito, Ae. vexans arabiensis, has the potential to be an important epidemic and epizootic vector because of the tremendous numbers of individual mosquitoes that are produced after a flooding rain. Whether or not RVFV is able to persist on the Arabian Peninsula is unknown. Clearly, vertical transmission of the virus in the epidemic mosquito vector would be an important factor to consider.

## References

[1] Centers for Disease Control and Prevention. Outbreak of Rift Valley fever—Saudi Arabia, August–October, 2000. MMWR Morb Mortal Wkly Rep. 2000;49:905–8. 11043643

[2] Peters CJ. Emergence of Rift Valley fever. In: Saluzzo JF, Dodet B, editors. Factors in the emergence of arboviruses. Paris: Elsevier; 1997. p. 253–64.

[3] White GB. Notes on a catalog of Culicidae of the Ethiopian region. Mosquito Systematics. 1975;7:303–44.

[4] Miller BR, Nasci RS, Lutwama JJ, Godsey MS, Savage HM, Lanciotti RS, First field evidence for natural vertical transmission of West Nile virus in Culex univittatus complex mosquitoes from Rift Valley Province, Kenya. Am J Trop Med Hyg. 2000;62:240–6. 10813479 10.4269/ajtmh.2000.62.240

[5] Swofford DL. PAUP*:Phylogenetic Analysis Using Parsimony (*and other methods). Version 4.08b. Sunderland (MA): Sinauer Associates; 1998.

[6] Sall AA, de A. Aanotto PM, Sene OK, Zeller HG, Digoutte JP, Thiongane Y, . Genetic reassortment of Rift Valley fever in nature. J Virol. 1999;73:8196–200. 10482570 10.1128/jvi.73.10.8196-8200.1999 PMC112837

